# Can chemotherapy alone eliminate the transmission of soil transmitted helminths?

**DOI:** 10.1186/1756-3305-7-266

**Published:** 2014-06-10

**Authors:** James E Truscott, T Déirdre Hollingsworth, Simon J Brooker, Roy M Anderson

**Affiliations:** 1 Department of Infectious Disease Epidemiology, London Centre for Neglected Tropical Disease Research, School of Public Health, Faculty of Medicine, St Marys Campus, Imperial College London, Praed Street, London W2 1PG, UK; 2 Warwick Mathematics Institute, University of Warwick, Coventry CV4 7AL, UK; 3 School of Life Sciences, University of Warwick, Coventry CV4 7AL, UK; 4 Department of Clinical Sciences, Liverpool School of Tropical Medicine, Pembroke Place, Liverpool L3 5QA, UK; 5 Faculty of Infectious and Tropical Diseases, London School of Hygiene and Tropical Medicine, London, UK; 6 Kenya Medical Research Institute, Nairobi, Kenya

**Keywords:** Soil-transmitted helminths, Chemotherapy, Elimination, Mathematical modelling

## Abstract

**Background:**

Amongst the world’s poorest populations, availability of anthelmintic treatments for the control of soil transmitted helminths (STH) by mass or targeted chemotherapy has increased dramatically in recent years. However, the design of community based treatment programmes to achieve the greatest impact on transmission is still open to debate. Questions include: who should be treated, how often should they be treated, how long should treatment be continued for?

**Methods:**

Simulation and analysis of a dynamic transmission model and novel data analyses suggest refinements of the World Health Organization guidelines for the community based treatment of STH.

**Results:**

This analysis shows that treatment levels and frequency must be much higher, and the breadth of coverage across age classes broader than is typically the current practice, if transmission is to be interrupted by mass chemotherapy alone.

**Conclusions:**

When planning interventions to reduce transmission, rather than purely to reduce morbidity, current school-based interventions are unlikely to be enough to achieve the desired results.

## Background

Amongst the world’s poorest populations, funding for the control of soil transmitted helminths (STH) by mass or targeted chemotherapy has increased steadily in the past 10 years due to generous donations from international aid agencies in the richer countries, philanthropic organisations and pharmaceutical companies [1]. Stimulated by the World Health Organisation’s (WHO) 2020 Roadmap on the control of the Neglected Tropical Diseases (NTDs), the spirit of this expanded effort is captured in the London Declaration in January 2012 and the progress report one year later [2-4]. Many questions remain, however, about how best to deliver community based treatment programmes for STH infections to obtain the greatest impact. These include the following: who should be treated, how often should they be treated, how long should treatment be continued for, can treatment intervals be increased as worm loads fall, and can transmission be eliminated by repeated chemotherapy alone? [5,6]. To answer these questions, a detailed understanding of the transmission dynamics of the parasites is required.

**Figure 1:**
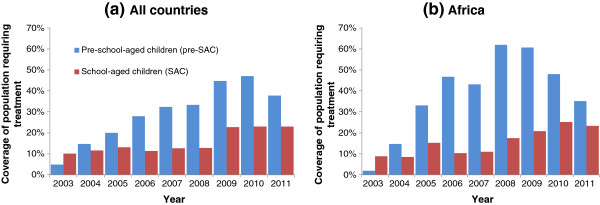
**Coverage of preventive STH chemotherapy in preschool-age children (Pre-SAC, blue) and school-age children (SAC, red), (a) internationally and (b) WHO African Region, calculated by WHO as the proportion of the total population of pre-SAC and SAC living in all the endemic areas in a country which require preventive chemotherapy for STH by year, 2003–2011**[1]**.**

Current guidelines for population-based treatment for STH infections focus on the treatment of pre-school-aged children (pre-SAC) between the ages of 2 to 4 years of age and school-aged children (SAC) between the ages of 5 to 14 years [7,8], due to measured health benefits and the low cost and simplicity of child health days and school health programmes used to deliver treatment [9,10]. The impact of increased funding on coverage in these age groups is illustrated in Figure 1, which shows high coverage amongst pre-school-aged children increasing coverage amongst school-aged children. The design and evaluation of many control programmes is based on measures of the prevalence (fraction infected [11,12]) and intensity of infection (worm loads or parasite egg concentrations in human faeces as a surrogate of worm load [13,14]). In this paper, we use a mathematical model to investigate the possibility of local elimination of parasites within a community by regular chemotherapeutic interventions and its dependence on the underlying strength of transmission and effective treatment coverage in the key age-groups of pre-SAC, SAC and adults.

## Methods

The mathematical model used describes the evolution of the parasite distribution in different host age groups and the impact of periodic chemotherapy on host burdens, incorporating the key epidemiological and biological processes influencing transmission. Building on past research [15,16], it includes the observed features of sexual reproduction by the dioecious helminths, heterogeneity in exposure to infection by host age, variation in the intensity of transmission in different human communities, aggregated distributions of worm numbers per host and a decline in fecundity as a function of worm burden (density dependence) [16-18]. The dynamics of transmission under repeated rounds of treatment is examined for the three main intestinal nematodes, *Ascaris lumbricoides*, *Trichuris trichuria* and hookworms (*Necator americanus* and *Ancylostoma duodenale*). The model is described in detail in the Additional file 1 available online.

Although a full age distribution is embedded in the model, we employ the key age groupings described above to define intervention coverage levels and illustrate their effect. These are infants (0–1 years of age) who cannot be treated under current licensure of the main anthelmintic drugs in wide use (e.g. albendazole and mebendazole), pre-school aged children (pre-SAC, 2–4 years of age), school aged children (SAC, 5–14 years of age) and adults (15+ years of age). Varying combinations of the fraction treated in each age grouping, treatment frequency and duration of treatment are explored. The fraction in each grouping effectively treated is a product of the fraction given treatment and drug efficacy (defined as the proportion of worms expelled). Within the current model, these two aspects of treatment are inseparable, and coverage of the population is represented as a proportion of worms treated. Drug efficacy is typically in the region of 90% or more for *Ascaris* and hookworms, but somewhat less for *Trichuris*[19-22]. It should be noted that the fraction treated is effectively chosen at random from the subpopulation. This model does not address systematic non-compliance.

The life cycles of these parasites involve free living stages that are passed in the faeces of the human host and mature to infective stages in the external habitat (eggs for *Ascaris* and *Trichuris* and larvae for hookworms). The infective stages of the parasite in the environment are represented in the model by a common pool of infectious material. The life span of these stages is typically weeks to months under favourable environmental conditions, and they are excreted in very large numbers [23-26]. Although this duration is short by comparison with adult worm life expectancies in the human host, infectious material in the environment acts as a reservoir which is unaffected by chemotherapy and can play a significant role in the dynamics of treatment. Dynamics of a range of parasites within the host population can be represented by the same model, with distinct parameter ranges for different species (See Additional file 1: Table S1).

**Figure 2:**
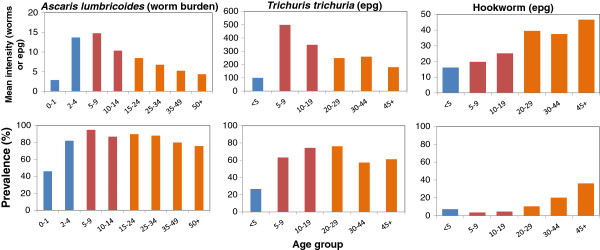
**Age-intensity profiles for mean intensity of infection (top row) and prevalence (%, second row) for the three major soil transmitted helminths; *Ascaris *[**[27]**] (left column), *Trichuris *[**[28]**] (middle column) & hookworm [**[29]**] (right column).** Colours indicate age-group.

Different age groups are thought to both contribute to, and be exposed to, this infective pool to varying degrees. An indication of this is provided by the changes in the intensity of infection by age; the patterns are typically convex for *Ascaris* and *Trichuris*, but continue to rise for hookworms as individuals age [27-29] (Figure 2). The respective roles of age related exposure to infection versus acquired immunity remains uncertain, but rapid re-infection by all three parasites post treatment points to the former as the main driver of age-intensity of infection profiles. On this basis, MCMC methods [30] are employed to fit the model to these age related patterns of infection, to estimate both transmission intensity (measured by the basic reproductive number R_0_ - the average number of offspring produced by one female worm that survive to reproductive maturity) and age related exposure. We have endeavoured to choose typical or characteristic infection profiles for the parasite species investigated in the hope that our results will be broadly applicable.

## Results

In the absence of regular treatment, the model exhibits two types of stable behaviour; either the endemic state in which the age profile of infection intensity matches those illustrated in Figure 2, for the respective parasite, or the parasite-free or elimination state. These two states are separated by a ‘breakpoint’ in transmission (created by sexual reproduction which necessitates finding a mate of the opposite sex) close to which parasite population growth is greatly restricted and below which the population decays to extinction [17]. It is possible for a single round of treatment to reduce parasite burdens in the population to a level from which they cannot recover. However, if a single round of treatment can reduce parasite populations to low levels close to the breakpoint, recovery of the parasites may be insufficient to prevent subsequent rounds from driving the population below the break-point. Hence, for a given frequency of treatment and parameterization of the model, we can define *critical* distributions of treatment among the host population that will eventually lead to elimination of the parasite from the population. Using the parameter estimates for the key epidemiological and biological processes defined in the Additional file 1: Table S2, treatment outcomes are explored for a wide range of effective treatment scenarios for the three major age groupings that can be treated (infants cannot be treated). Three different settings are explored, where transmission intensity is low (R_0_ = 2), medium (R_0_ = 3) and high (R_0_ = 5). These stratifications differ somewhat from WHO definitions of low, medium and high transmission settings for reasons defined in the Additional file 1, but related to the fact that prevalence, the WHO favoured outcome statistic, is a poor measure of the intensity of infection due to the highly non-linear relationship between these two epidemiological quantities which are drawn from aggregated distributions (variance much bigger than the mean in value) such that large changes in intensity may result in only small changes in prevalence [17].

**Figure 3:**
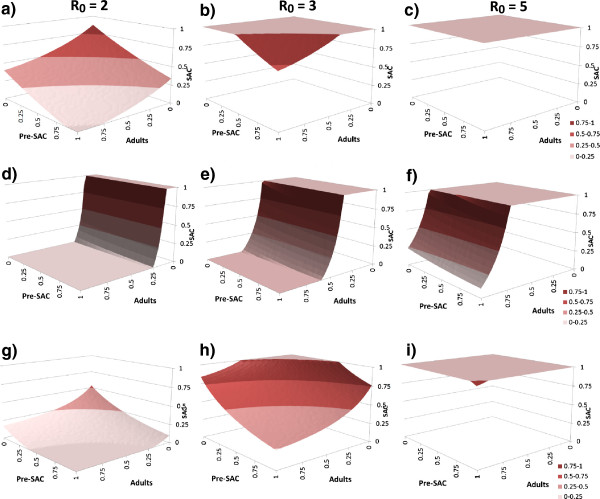
**The critical treatment surfaces for *Ascaris* (row 1: a, b, c, and 3: g, h, i) and hookworm (row 2: d, e, f).** Reproductive number R_0_ is 2, 3 and 5 in columns 1 (a, d, g), 2 (b, e, h) and 3 (c, f, i), respectively. In rows 1 (a, b, c) and 2 (d, e, f), treatment is yearly while in row 3 (g, h, i), treatment is every 6 months. Model parameters as in Additional file 1: Table S2. The proportion treated effectively (proportion treated x drug efficacy) in each age grouping (pre-SAC, SAC & Adults) must lie above the ‘critical’ surface in the 3D plots (at the plateau of 1.0 all must be treated effectively) for eradication to occur. The colours indicate coverage in SAC.

**Figure 4:**
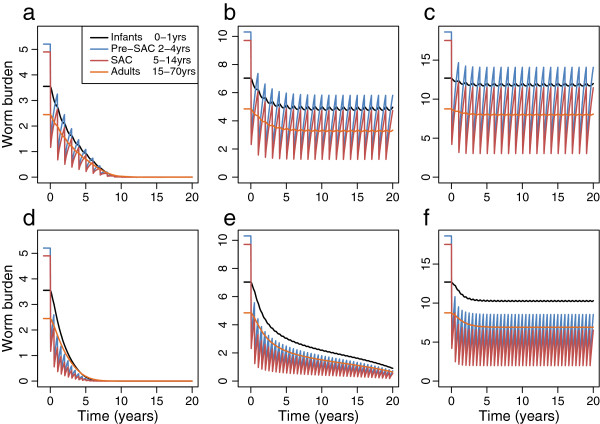
**Numerical solutions of the model for different transmission settings (R**_**0 **_**of 2 (a, d), 3 (b, e) and 5 (c, f)) and yearly (a, b, c) or twice-yearly (d, e, f) treatment with 80% treatment in Pre-SAC and SAC.** The mean worm burden in Infants (black), Pre-SAC (blue), SAC (red) and Adults (orange) is shown reducing over time. Other parameters as in Additional file 1: Table S2.

The results are presented in two formats; namely, a three dimensional surface of the effective treatment combinations of pre-SAC, SAC and adults that results in crossing the critical treatment surface to extinguish parasite transmission (values equal to or above the surface lead to long term extinction), and time series of repeated treatment of different proportions of the three treatment age groupings to determine how long treatment must continue to the cessation of transmission. These are presented in Figures 3 and 4 respectively for the two formats. The parasite with the greatest R_0_ value determines the intensity, frequency and duration of treatment required. Graphs are for *Ascaris* and hookworm only, since *Trichuris* exhibits very similar patterns to *Ascaris* in terms of its age-intensity profile, excepting drug efficacy is somewhat lower.

A series of general conclusions emerge from these calculations. Annual treatment of only school aged children will rarely extinguish transmission, except in very low *Ascaris* and *Trichuris* transmission settings (R_0_ = 2 or less). For example, when R_0_ = 2, SAC need to be treated with an overall effectiveness better than 80% for elimination to be achieved (Figure 3a). That is, the product of the fraction of the SAC population covered with the efficacy of the drug must exceed 80%. Once R_0_ > 2 some of either adults, pre-SAC or both must be treated as well at high coverage levels (Figure 3b,c,e,f). In most settings where *Ascaris* and/or *Trichuris* are prevalent, R_0_ exceeds 2.5 in value [31,32]. Treating both SAC and pre-SAC, as sometimes carried out by control programmes, with high coverage (>80%) results in good suppression but the ‘breakpoint’ is not crossed. Treating only SAC works even less well if the dominant parasite is hookworm, since most infection and production of infective stages occurs in the adults (Figures 2 and 3). In this case, the coverage of the pre-SAC population has almost no impact on the effect of treatment. A large proportion (>50% of the adults must be treated to interrupt transmission in moderate to high transmission settings (Figure 3d,e,f). In medium and high transmission settings, if either *Ascaris* or *Trichuris* are the most prevalent infections, the pre-SAC and SAC individuals appear to contribute most to transmission (based on the MCMC estimates of age related infection rates from age-intensity profiles). In these circumstances pre-SAC and adults must be treated as well as SAC with coverage dependent on the value of R_0_. For very high values (e.g. R_0_ = 5, Figure 3c) coverage must be above 80% for all age groupings if treatment is annual.

Current guidelines recommend that treatment is administered every 12 months in low-medium transmission settings and every 6 months in high transmission settings [7,8]. The model results show that the target coverage level can be reduced if treatment is administered more frequently such as every 6 months. This is shown for *Ascaris* in Figure 3g, where in low to medium transmission settings the critical treatment threshold can be attained by only treating SAC (at above 70% for R_0_ = 2 and above 90% for R_0_ = 3). In high settings, treatment levels that do not trigger crossing the treatment threshold surface will have to be sustained indefinitely to avoid a return to pre-control levels. Increasing treatment frequency (every 4 months) and/or changes in behaviour and sanitation that restrict contamination of the environment with infective stages (and hence lower the value of R_0_) will be required if elimination of transmission is to occur.

The time span over which elimination can be achieved is very sensitive to the details of the intervention and the transmission settings. Figure 4 shows the response of parasite burden in the four age categories to 80% effective coverage for preSAC and SAC. For low values of R_0_, and yearly treatment for *Ascaris*, the threshold can be crossed with 10 years at high (80%) treatment coverage (Figure 4a). For 6-monthly treatment, the time is reduced to approximately 6 years (Figure 4d). Treatment at this frequency also allows elimination at R_0_ = 3. However, the process takes longer than 20 years. As shown in Figure 3, elimination is generally not possible for medium to high transmission. Figure 4b,c,f shows that a new ‘settled’ worm burden has been reached. In general, relaxation of treatment in overall efficacy or frequency leads to a recovery of worm burden in the population. However, preliminary results suggest that if repeated treatment can bring community worm burden sufficiently close to critical level at which elimination is inevitable, it may be possible to relax the intervention without significant recovery for the parasite. This phenomenon is the object of further work.

## Discussion

The surfaces shown in Figure 3 clearly show that elimination of Ascaris purely through school-based deworming is not possible except where transmission settings are at their lowest. Some additional coverage of pre-school children and/or adults is also required. For hookworm, some coverage of adults is essential. The surfaces indicate what ranges of effective coverage will allow elimination but not how long the process may take. As Figure 4 indicates, the elimination goal may be reached in 5–6 years or over several decades and can be very sensitive to the details of treatment coverage and the dynamics of the parasite in the host population.

The optimal approach to treating age groups other than school-age children depends not only on the epidemiological impact but also the practicality and cost-effectiveness of alternative approaches. Perhaps the longest running community-based helminth control programme that treats across all age groups is the African Programme for Onchocerciasis Control (APOC), which has helped countries create a community-directed treatment (CDT) strategy involving both community-directed drug distributors and extending and strengthening health systems [33-36]. The CDT approach is also employed by lymphatic filariasis (LF) control programmes in Africa which administer albendazole and ivermectin (or diethylcarbamazine, DEC) to entire populations aged 2 years and above in LF endemic areas [37]. In addition to the role of community drug distributors, there is a potential for community health workers to deliver treatment, as recently demonstrated for schistosomiasis control in western Kenya [38] and STH control in Ethiopia [39]. A randomised community evaluation in Kenya is currently underway to evaluate the impact and cost-effectiveness of school- versus community-based delivery of albendazole on the transmission of STH in settings with different levels of transmission and species mix. A qualitative evaluation nested within this study will also investigate the acceptability and feasibility of using alternative delivery platforms.

In many poor rural areas of sub-Saharan Africa and southeast Asia long-term commitments to frequent treatment will be difficult to achieve and, as we demonstrate here, in high transmission settings, treatment alone will not interrupt transmission. In such settings, there is a basic requirement to improve hygienic behaviour and access to improved drinking water and sanitation (WASH). The potential benefits of increased access for reducing the risk of STH has been recently highlighted by a systematic review of the associations between STH and water and sanitation which found that people with access to improved water and sanitation facilities were half as likely to be infected with any STH species, with the strongest associations for access to sanitation and use of soap [40]. Levels of access to improved drinking water and sanitation varies greatly within countries [41], and understanding the geographical inequality in access can help identify areas where treatment can and cannot break transmission. In areas where access is relatively high, there is a real potential to reduce and/or interrupt transmission; in areas where access to water and sanitation remains low, the priority should be on increasing access. Ongoing work is investigating the influence of access to water and sanitation as well as other contextual and programmatic factors on the feasibility of interrupting transmission and mapping those areas where this might be possible.

Our model provides insight into optimal approach to the control of STH, but our approach is not without its limitations. There remains much uncertainty about the nature of transmission dynamics between individuals. While infectious contact rates can be inferred from the baseline infection profile, little can be said about the nature of the infection process or whether a single environmental reservoir is the best model. We have chosen to use the ‘trickle’ model of the infection process rather than the ‘clumping’ model, primarily due to its convenience within a deterministic model paradigm [42]. Other work has shown that the clumping model can have a significant impact on the model’s response to treatment and its sensitivity to parameter values [42,43]. To resolve these questions will require longitudinal treatment trials, recording data at multiple time-points from both treated and untreated age-groups over 3–5 years. Such trials are currently in progress in Kenya.

Details of model structure and precise parameter values are only important if the behaviour modelled is sensitive to them. In the current paper, we have not included a formal sensitivity analysis, but numerical investigations indicate that the parameter groupings we have focused on (worm lifespan, *R*_*0*_ and proportion and frequency of effective treatment) are those to which the model is most sensitive. The aggregation of the worms among hosts (*k*), timescale of infectious material in the environment (*μ*_*2*_) and population demography do not have a strong effect on the results. We investigate assumptions around the deposition of eggs and acquisition of infection in different ages in a follow-up publication [44].

The current model also does not take into account systematic non-compliance with repeated treatments, which may create a reservoir of transmission that is difficult to reach. It is probable that certain groups are more likely to systematically reject treatment and extra effort is required to target these groups [45]. This phenomenon is the subject of ongoing work.

Global STH control efforts are currently focused on scaling up treatment coverage in school-aged children to 75%, a goal which is justified on the health and education benefits of treating this age group and which is achievable given current drug donation programmes and increased funding. The achievement of this coverage goal should remain the priority of national governments and the international community. However, there is an additional potential to interrupt transmission by expanding treatment to additional age groups and increasing treatment frequency in some settings. Work is underway to validate our models with available data and to evaluate the impact of different treatment strategies in a graded series of randomised evaluations in contrasting transmission settings.

## Conclusion

This modelling analysis has shown how the possibility of breaking the parasite transmission cycle depends on the age groups treated, the coverage and efficacy of treatment and the species in question. Our results strongly indicate that when planning to reduce or eliminate transmission, rather than simply reduce morbidity, treatment of school-age children will be insufficient; some treatment of pre-school-age children or adults is necessary, depending on the transmission setting and worm species.

## Competing interests

Roy M. Anderson is non-executive member of the board of GlaxoSmithKline (GSK). GlaxoSmithKline played no role in study design, data collection and analysis, decision to publish, or preparation of the manuscript.

## Authors’ contributions

RMA, TDH & JT contributed equally to the design of the study, equation formulation, analysis and writing of the paper. SB contributed to the design of the study and writing of the paper. All authors read and approved the final version of the manuscript.

## Supplementary Material

Additional file 1 **Model description and parameters including Figure S1 Approximate relationships for soil-transmitted helminths between prevalence (as a proportion) and the mean worm burden, and prevalence and the basic reproductive number R**_**0 **_**(simple relationship – no mating function, no age structure).** Click here for file
