# Supplementary material for: Can chemotherapy alone eliminate the transmission of soil transmitted helminths?
Source: Parasit Vectors. 2014 Jun 10;7:266. doi: 10.1186/1756-3305-7-266 (PMC4079919; doi:10.1186/1756-3305-7-266)
Supplement: Additional file 1 — Model description and parameters including Figure S1 Approximate relationships for soil-transmitted helminths between prevalence (as a proportion) and the mean worm burden, and prevalence and the basic reproductive number R 0 (simple relationship – no mating function, no age structure). [file 1756-3305-7-266-S1.docx]

**Can chemotherapy alone eliminate the transmission of soil transmitted helminths?**

James E. Truscott^1^, T. Déirdre Hollingsworth ^2,3,4^, Simon J. Brooker ^5,6^, Roy M. Anderson ^1^

^1^London Centre for Neglected Tropical Disease Research, Department of Infectious Disease Epidemiology, School of Public Health, Faculty of Medicine, St Marys Campus, Imperial College London, Praed Street, London W2 1PG,

^2^Mathematics Institute, University of Warwick, Coventry, CV4 7AL, UK

^3^School of Life Sciences, University of Warwick, Coventry, CV4 7AL, UK

^4^Department of Clinical Sciences, Liverpool School of Tropical Medicine, Pembroke Place, Liverpool, L3 5QA , UK

^5^Faculty of Infectious and Tropical Diseases, London School of Hygiene and Tropical Medicine, London, United Kingdom

^6^Kenya Medical Research Institute–Wellcome Trust Research Programme, Nairobi, Kenya

**Additional file – model description and parameters**

**Mathematical model**

We employ a deterministic model to represent the dynamics of worm burden in 4 contiguous age classes; infants (0-2 years of age), pre-school age children (2-4 years of age), school aged children (5-14 years of age), and adults (all >15 years old). Previous work [^1^](#_ENREF_1) has analysed the dynamics of 2 age class models (less than and greater than 15 years) under regular treatment. However, in the present case, the short age ranges that are a feature of this model are comparable to worm lifespans (1-2.5 years). Hence we use an explicitly age-structured model and superimpose our desired age structure on it.

The fundamental model used to describe the evolution of the worm burden of individuals of age a and the quantity of infectious material in the environment is taken from Anderson and May [^2^](#_ENREF_2).

The variable $M(t,a)$describes the mean worm burden of a host of age *a* at time *t*. The underlying distribution is assumed to be negative binomial. The variable *L(t)* represents the concentration of infectious material in the environment at time *t*. The function *f(.)* captures the density dependence of fecundity and is a reduction factor accounting for the effects of sexual reproduction of worms in the host[^2^](#_ENREF_2).

Here, k is the shape parameter of the assumed negative binomial distribution of worms among hosts (varying inversely with the degree of clumping) and z is the density-dependent fecundity parameter (it’s assumed that the model is in terms of female worms and that the effect of fecundity is dependent on the host burden of *female* worms).

The effects of host behaviour are encapsulated in the age-dependent parameters $\rho(a)$, which govern what fraction of an individual’s egg output enters the reservoir, and the $\beta(a)$, which govern the degree of exposure of the various age groups to the reservoir. Only the relative values of these parameter vectors are important, as the absolute size can be absorbed into total egg deposition rate *ψ*. In the simulations used in this paper, we have used a demographical profile to match the population of Uganda[^3^](#_ENREF_3). The demography of the host population is described by the survival function, *H(a)*, representing the probability for an individual to reach age *a*. The survival function is related to the mortality, *μ(a)*, through

$$\ln(H(a))=-\int_{t=0}^{a} \mu(t)dt$$

The parameter $H_{T}=\int H(a)da$.For this model, the value of *R_0_* is given by the expression

$$R_{0}=\frac{\psi\lambda z}{H_{T}\mu}\int_{a=0}^{a_{\max}} \frac{\beta(a)}{S(a)}\int_{\tau=a}^{a_{\max}} \rho(\tau)H(\tau)S(\tau)d\tau da$$

The parameter *S(a)* is the survival function for a worm recruited into a host at birth.

$$\ln(S(a))=-\int_{t=0}^{a} (\mu(t)+\sigma)dt$$

In practice, we use a discretized version of the evolution equations with separate equations for the worm burden in annual age classes. The model has the form

where $1\leq i\leq N$. The parameter $\Delta_{i}$is the width of the i^th^ age class. We use annual age classes, so $\Delta_{i}=1$and *N*=70. Age dependent parameters, such as $\beta$ and *ρ*, are discretized into N values, one for each age class. Similarly, the expression for *R_0_* is approximated by summations in place of integrals. Age-dependent parameters have distinct values within each of the broad age classes described above (Pre-SAC, SAC, Adult). Hence $\beta_{0},\beta_{1},\beta_{2},\beta_{3}$ and $\beta_{4}$all have the $\beta$ value assigned to the Pre-SAC age group. Since only the relative values of $\beta$ and *ρ* are important, we arbitrarily define $\beta$ and *ρ* values to be 1 for the SAC group.

Treatment efficacy is treated in the same fashion, with distinct levels of treatment in each of the three age categories giving an efficacy, γ_i_, in the i^th^ annual age group. Treatment is applied at regular intervals and reduces the worm burden in a class by a factor γ_i_. To ascertain whether a particular treatment age profile and interval resulted in eradication of the parasite, the model was run from its treatment-free equilibrium through a sequence of treatment intervals lasting 20 years. For a given level of treatment in the pre-SAC and adult age groups, the bisection algorithm was used to identify the lowest level in treatment for the SAC group that resulted in long-term eradication.

**Parameter estimates**

The majority of parameter values for the model described above were taken from sources in the literature. Parameter estimates are quite sparse due to the difficulties of measurement. Table S1 gives a brief survey of values for k, z and R_0_ across different studies and species. While variability is wide, there are clear differences between species. The values used in our models are identified in Table S2. However, data for the age-specific contact rate of hosts with the infectious reservoir ($\beta$) and age-specific contribution of hosts to the reservoir ($\rho$) are unknown. These were estimated by fitting the model to worm burden age profile data [^4^](#_ENREF_4),[^5^](#_ENREF_5). The age-dependent variable, M, in our model represents the mean of a negative binomial distribution, making it straight-forward to construct a likelihood for a given set of data. In each case, other parameters were chosen to match species natural history and the survival profile of the host population in the area of the study and at the time it was carried out. Using Monte-Carlo Markov (MCMC) chain methods, we identified the maximum likelihood estimators for *R_0_* and $\beta$ in the three observed age categories. The MCMC chain was constructed using the MCMC package in R (version 2.15.1). The values of $\rho$ have no effect on the shape of the endemic worm burden age profile, so we assume that the rate of contact with the infectious reservoir is proportional to the contribution to the reservoir of a given age class: hence $\beta_{i}\propto\rho_{i}$ for observed age class, *i*.

**WHO definitions of low, medium and high transmission settings**

WHO definitions of low, medium and high transmission settings for STH are represented for prevalence in Fig S1. Intensity boundaries have also been specified in terms of eggs per gram of faeces (epg) separately for each of the three major STH nematodes. They are as follows: *Ascaris lumbricoides*, 1-4999epg (low), 5000-49999epg (medium), ≥ 50000epg (high)**;** *Trichuris trichuria*, 1-999epg (low), 1000-9999epg (medium), ≥ 10000epg (high); Hookworms, 1-1999epg (low), 2000-3999epg (medium), ≥4000 (high). The reasons (either clinical in terms of morbidity or epidemiological) lying behind these intensity boundaries are unclear. In an epidemiological context, it may be more appropriate to define low, medium and high transmission settings in terms of R_0_ values as adopted in this paper.

**
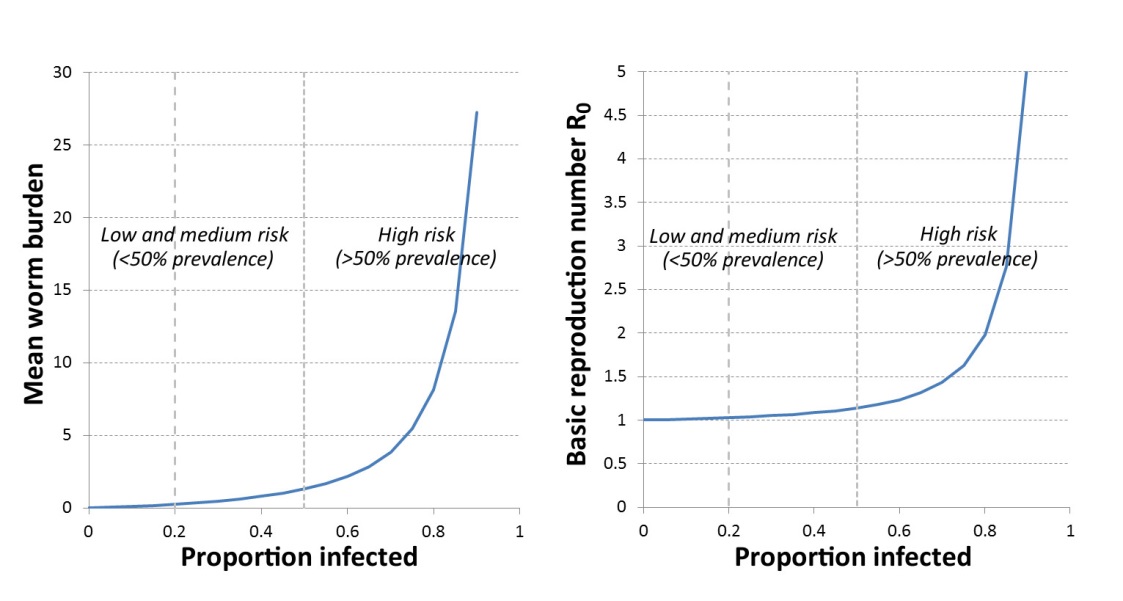
**

**Figure S1** Approximate relationships for soil-transmitted helminths between prevalence (as a proportion) and the mean worm burden, and prevalence and the basic reproductive number R_0_ (simple relationship – no mating function, no age structure). The vertical bars are the delineators between the WHO definitions based on prevalences in low, medium and high transmission areas. Note that the definition of high as a prevalence >50% covers R_0_ values in excess of 2, and low and medium transmission areas are for R_0_ values between 1 and 2. In the analyses presented in the main text, low transmission areas are defined as R_0_=1 to 2, medium as R_0_=3 to 4 and high as R_0_ ≥ 5.

**References**

1 Truscott, J. E., Hollingsworth, T. D. & Anderson, R. M. The interruption of the transmission of soil transmitted helminths by repeated mass chemotherapy of school aged children: transmission thresholds and the significance of non-random contact with infective stages. *in preparation* (2013).

2 Anderson, R. M. & May, R. M. Helminth infections of humans - mathematical models, population dynamics, and control. *Adv Parasitol* **24**, 1-101 (1985).

3 Pullan, R. L., Kabatereine, N. B., Quinnell, R. J. & Brooker, S. Spatial and genetic epidemiology of hookworm in a rural community in Uganda. *PLoS Negl Trop Dis* **4**, e713, doi:10.1371/journal.pntd.0000713 (2010).

4 Bradley, M., Chandiwana, S. K., Bundy, D. A. & Medley, G. F. The epidemiology and population biology of Necator americanus infection in a rural community in Zimbabwe. *Trans R Soc Trop Med Hyg* **86**, 73-76 (1992).

5 Elkins, D. B., Haswell-Elkins, M. & Anderson, R. M. The epidemiology and control of intestinal helminths in the Pulicat Lake region of Southern India. I. Study design and pre- and post-treatment observations on Ascaris lumbricoides infection. *Trans R Soc Trop Med Hyg* **80**, 774-792 (1986).

6 Holland, C. V. *et al.* The epidemiology of Ascaris lumbricoides and other soil-transmitted helminths in primary school children from Ile-Ife, Nigeria. *Parasitology* **99 Pt 2**, 275-285 (1989).

7 Croll, N. A., Anderson, R. M., Gyorkos, T. W. & Ghadirian, E. The population biology and control of Ascaris lumbricoides in a rural community in Iran. *Trans R Soc Trop Med Hyg* **76**, 187-197 (1982).

8 Hall, A., Anwar, K. S., Tomkins, A. & Rahman, L. The distribution of Ascaris lumbricoides in human hosts: a study of 1765 people in Bangladesh. *Trans R Soc Trop Med Hyg* **93**, 503-510 (1999).

9 Thein, H., Than, S., Htay Htay, A., Myint, L. & Thein Maung, M. Epidemiology and transmission dynamics of Ascaris lumbricoides in Okpo village, rural Burma. *Trans R Soc Trop Med Hyg* **78**, 497-504 (1984).

10 Bundy, D. A., Cooper, E. S., Thompson, D. E., Didier, J. M. & Simmons, I. Epidemiology and population dynamics of Ascaris lumbricoides and Trichuris trichiura infection in the same community. *Trans R Soc Trop Med Hyg* **81**, 987-993 (1987).

11 Martin, J., Keymer, A., Isherwood, R. J. & Wainwright, S. M. The prevalence and intensity of Ascaris lumbricoides infections in Moslem children from northern Bangladesh. *Trans R Soc Trop Med Hyg* **77**, 702-706 (1983).

12 Chai, J. Y., Kim, K. S., Hong, S. T., Lee, S. H. & Seo, B. S. Prevalence, worm burden and other epidemiological parameters of Ascaris lumbricoides infection in rural communities in Korea. *Kisaengchunghak Chapchi* **23**, 241-246, doi:198512241 [pii] (1985).

13 Quinnell, R. J. *et al.* Reinfection with hookworm after chemotherapy in Papua New Guinea. *Parasitology* **106**, 379-385 (1993).

14 Haswell-Elkins, M. R., Elkins, D. B., Manjula, K., Michael, E. & Anderson, R. M. An investigation of hookworm infection and reinfection following mass anthelmintic treatment in the south Indian fishing community of Vairavankuppam. *Parasitology* **96 ( Pt 3)**, 565-577 (1988).

15 Anderson, R. M. & Schad, G. A. Hookworm burdens and faecal egg counts: an analysis of the biological basis of variation. *Trans R Soc Trop Med Hyg* **79**, 812-825 (1985).

16 Hoagland, K. E. & Schad, G. A. Necator americanus and Ancylostoma duodenale: life history parameters and epidemiological implications of two sympatric hookworms of humans. *Experimental parasitology* **44**, 36-49 (1978).

17 Nawalinski, T. & Roundy, L. M. Intestinal parasitism in a Kampong on Pulau Pangkor, West Malaysia. *Southeast Asian J Trop Med Public Health* **9**, 440-441 (1978).

18 Ye, X. P., Wu, Z. X. & Sun, F. H. The population biology and control of Necator americanus in a village community in south-eastern China. *Annals of tropical medicine and parasitology* **88**, 635-643 (1994).

19 Bundy, D. A. *et al.* Predisposition to Trichuris trichiura infection in humans. *Epidemiol Infect* **98**, 65-71 (1987).

20 Yadav, A. K. Development and survival of Ascaris lumbricoides eggs under the high-rainfall and humid conditions prevailing in Meghalaya, India. *Proc. zool. Soc. Calcutta* **56**, 109-112 (2003).

21 Larsen, M. N. & Roepstorff, A. Seasonal variation in development and survival of Ascaris suum and Trichuris suis eggs on pastures. *Parasitology* **119 ( Pt 2)**, 209-220 (1999).

22 Anderson, R. M. The dynamics and control of direct life cycle helminth parasites. *Lecture Notes in Biomathematics* **39**, 278-322 (1980).

23 Udonsi, J. K. Experimental and field studies on the viability of eggs, and the infectivity of third-stage larvae of the human hookworm Necator americanus recovered from the creek waters of the Niger Delta, Nigeria, and their epidemiological significance. *Parasitology* **96 ( Pt 1)**, 111-117 (1988).

24 Croll, N. A. & Matthews, B. E. Activity, ageing and penetration of hookworm larvae. *Parasitology* **66**, 279-289 (1973).

| **Parasite** | **R_0_** | **k** | **z** | **Adult worm life expectancy** | **Region** | **Reference** |
| --- | --- | --- | --- | --- | --- | --- |
| *Ascaris* |  | 0.81 | 0.968 | 1 year | India | Elkins et al, [^5^](#_ENREF_5) |
| *Ascaris* |  |  | 0.927 |  | Nigeria | Holland et al, [^6^](#_ENREF_6) |
| *Ascaris* | 4-5 | 0.57 | 0.991 |  | Iran | Croll et al, [^7^](#_ENREF_7) |
| *Ascaris* |  | 0.6-0.7 |  |  | Bangladesh | Hall et al, [^8^](#_ENREF_8) |
| *Ascaris* | 1-3 | 0.46 |  |  | Myanmar | Thein-Hliang et al, [^9^](#_ENREF_9) |
| *Ascaris* |  | 0.59 |  |  | St Lucia | Bundy et al, [^10^](#_ENREF_10) |
| *Ascaris* | 1-2 | 0.44 |  |  | Bangladesh | Martin et al, [^11^](#_ENREF_11) |
| *Ascaris* |  | 0.36 to 0.54 |  |  | South Korea | Chai et al, [^12^](#_ENREF_12) |
| *Ascaris* |  | 0.54 |  |  | Many countries | Guyatt et al, 1990 |
| *Ascaris* |  | - | 0.992 |  | Malaysia | Sinniah et al, 1983 |
| *Ascaris* |  | 0.2-0.5 |  |  | Japan |  |
| Hookworm |  | 0.45 |  |  | Papua New Quinea | Quinnell et al, [^13^](#_ENREF_13) |
| Hookworm |  | 0.35 | 0.92 |  | Zimbabwe | Bradley et al.[^4^](#_ENREF_4) |
| Hookworm |  | 0.24 |  |  | India | Haswell-Elkins et al, [^14^](#_ENREF_14) |
| Hookworm |  | 0.63 |  |  | India | Anderson & Schad [^15^](#_ENREF_15) |
| Hookworm  *Ancylostoma*  *Necator* | 2-3 | -  0.03-0.6 |  | 1 year  3-4 years | India | Hoagland & Schad, [^16^](#_ENREF_16)  ; Nawalinski et al, [^17^](#_ENREF_17) |
| *Necator* |  | 0.16-0.24 |  |  | India | Haswell-Elkins et al, [^4^](#_ENREF_4) |
| *Necator* |  | 0.05-0.4 |  |  | Taiwan | Anderson, 1980 |
| *Necator* | 3-4 |  |  |  | China | Ye et al, [^18^](#_ENREF_18) |
| *Necator* | 2 | 0.35 |  |  | Zimbawe | Bradley et al, [^4^](#_ENREF_4) |
| *Trichuris* | 8-10 | 0.2-0.4 |  |  | St Lucia | Bundy et al, [^10^](#_ENREF_10) |
| *Trichuris* | 4-6 |  |  |  | Jamaca | Bundy et al, [^19^](#_ENREF_19) |

Table S1: Literature survey of estimates for key epidemiological parameter values.

| **Parameter** | **Value** | **Source** |
| --- | --- | --- |
| **Ascaris Lumbricoides** |  |  |
| Aggregation parameter, *k* | 0.7 | Elkins et al [^5^](#_ENREF_5)* |
| Density dependent fecundity, *z* | 0.93 | Holland et al [^6^](#_ENREF_5)* |
| Worm lifespan in human | 1 year | Croll [^7^](#_ENREF_7) |
| Half-life of infective material in the environment, *µ_2_* | 1-2 months | Yadav et al [^20^](#_ENREF_20), Larsen and Roepstorff^[21](#_ENREF_21)^ |
| Relative values for *β* and *ρ*  [Pre-SAC, SAC, Adults] | [1, 1, 0.5] |  |
|  |  |  |
| **Hookworm** |  |  |
| Aggregation parameter, *k* | 0.35 | Bradley et al. [^4^](#_ENREF_4) |
| Density dependent fecundity, *z* | 0.92 | Bradley et al. [^4^](#_ENREF_4) |
| Worm lifespan in human | 2 years | Anderson[**^22^**](#_ENREF_22) |
| Half-life of infective material in the environment, *µ_2_* | 1-2 months | Udonsi^[23](#_ENREF_23)^; Croll and Matthews[^24^](#_ENREF_24) |
| Relative values for *β* and *ρ*  [Pre-SAC, SAC, Adults] | [1.8, 1, 5.3] |  |
|  |  |  |
| **Demography** |  |  |
| Simulation host age profile | - | Pullan et al. [^3^](#_ENREF_3) |

Table S2: Model parameter values and sources. For sources marked with *, fitting was done to raw data by the authors.
